# Assessment of abdominal organ motion using cine magnetic resonance imaging in different gastric motilities: a comparison between fasting and postprandial states

**DOI:** 10.1093/jrr/rrz054

**Published:** 2019-08-24

**Authors:** Hotaka Nonaka, Hiroshi Onishi, Makoto Watanabe, Vu Hong Nam

**Affiliations:** 1 Department of Radiology, University of Yamanashi, Institutional address: 1110 Shimokato, Chuo City, Yamanashi, Japan; 2 Department of Radiology, Fujiyoshida Municipal Hospital, Institutional address: 6530 Kamiyoshida, Fujiyoshida City Yamanashi, Japan; 3 Department of Radiological Technology, Fujiyoshida Municipal Hospital, Institutional address: 6530 Kamiyoshida, Fujiyoshida City Yamanashi, Japan; 4 Department of Oncology and Nuclear Medicine, Hospital 175, Institutional address: 786 Nguyen Kiem Street, Ward 3, Go Vap District, Ho Chi Minh City, Viet Nam

**Keywords:** abdominal organ motion, gastric motility, cine magnetic resonance imaging, intra-fractional motion

## Abstract

This study assessed abdominal organ motion induced by gastroduodenal motilities in volunteers during fasting and postprandial states, using cine magnetic resonance imaging (cine-MRI). Thirty-five volunteers underwent cine-MRI while holding their breath in the fasting and postprandial states. Gastric motility was quantified by the amplitude and velocity of antral peristaltic waves. Duodenal motility was evaluated as the change of duodenal diameter. Abdominal organ motion was measured in the liver, pancreas and kidneys. Motion was quantified by calculating maximal organ displacement in the left–right, antero–posterior and caudal–cranial directions. Median antral amplitude and velocity in the fasting and postprandial states were 7.7 and 15.1 mm (*P* < 0.01), and 1.3 and 2.5 mm/s (*P* < 0.01), respectively. Duodenal motility did not change. Median displacement for all organs ranged from 0.9 to 2.9 mm in the fasting state and from 1.0 to 2.9 mm in the postprandial state. Significant increases in abdominal organ displacement in the postprandial state were observed in the right lobe of the liver, pancreatic head and both kidneys. Differences in the median displacement of these organs between the two states were all <1 mm. Although the motion of several abdominal organs increased in the postprandial state, the difference between the two states was quite small. Thus, our study suggests that treatment planning and irradiation need not include strict management of gastric conditions, nor the addition of excess margins to compensate for differences in the intra-fractional abdominal organ motion under different gastric motilities in the fasting and postprandial states.

## INTRODUCTION

Recently, high-precision radiotherapy, such as stereotactic body radiation therapy or intensity modulated radiation therapy, has become available to treat abdominal tumors. High-precision radiotherapy requires an accurate understanding of the intra-fractional motions of abdominal tumors and organs. Respiration contributes greatly to overall abdominal organ motion. Abdominal organ motion induced by respiration has been previously studied [1–3], and unreliable tumor dose and normal tissue volume due to respiration on treatment planning computed tomography has been reported [4, 5]. Following recent technological advances, cine magnetic resonance imaging (cine-MRI) has been proposed to monitor gastric or gastroduodenal motilities [6–11]; cine-MRI has been previously used for measurements of abdominal tumor motion that are primarily induced by respiration [12, 13]. However, there is a lack of focused studies of the abdominal motion induced by gastrointestinal motilities, the diet management needed to control such motion, and the internal margins required to take this motion into account when planning irradiation treatment of these organs. To the best of our knowledge, this is the first study of the relationship between intra-fractional abdominal organ motions, and gastroduodenal motilities.

The aim of this study was to determine whether gastroduodenal motilities influence the motion of upper abdominal organs such as the liver, pancreas and kidneys. To address this objective, we used cine-MRI to assess differences in the motion of upper abdominal organs in healthy volunteers during fasting and postprandial states.

## MATERIALS AND METHODS

### Volunteers and conditions of the stomach

An appropriate institutional review board approved this study. Written informed consent was obtained from each volunteer. A total of 35 (17 men and 18 women) volunteers were included in this study. Volunteers were excluded if they had an ongoing abdominal disease, a history of abdominal surgery and/or general contraindications for MRI. The median age of participants was 37 years old (range, 27–61) and the median body mass index was 22.2 kg/m^2^ (range, 17.8–35.4).

Volunteers fasted for a 5-h period with no food, then a 3-h period with no food or liquid, before their first MRI examination. Volunteers ingested jelly that was previously used to obtain clear images of the gastric wall and fold (360 mL, 360 kcal, Weider in jelly; Morinaga & Co, Tokyo, Japan; [8]) immediately following the first cine-MRI, then underwent the second cine-MRI in the postprandial state, 30 min after jelly ingestion.

### Cine-MRI

MRI examinations were performed in the supine position using a 1.5T MR scanner (Signa, HDxT, 1.5T, GE, Healthcare, Milwaukee, WI). A body array coil was not used in order to avoid abdominal compression. Cine-MRI was performed using a steady-state free precession sequence (fast imaging employing steady-state acquisition sequence: repetition time = 4.71 ms, echo time = 2.08 ms, flip angle = 40°, slice thickness = 5 mm, matrix = 288 × 192, field of view = 360 mm). A series of 24 images of the stomach, duodenum, liver, pancreas and kidneys was collected over a 20-s period. Breathing was held, following voice guidance, to eliminate abdominal organ motion induced by respiration.

### Assessment of gastroduodenal motilities and abdominal organ displacements

**Fig. 1.**
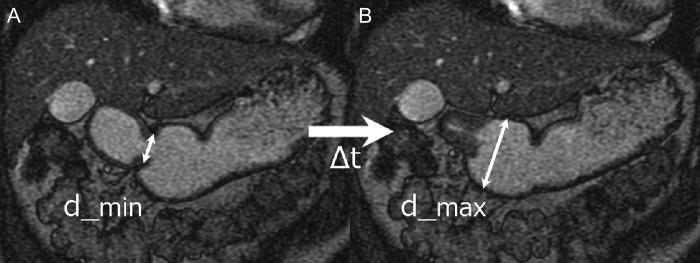
Oblique images of the gastric antrum with steady-state free precession sequence in a volunteer with minimum (**A**) and maximum (**B**) antral diameters of a peristaltic wave. Maximum diameter = d_max, minimum diameter = d_min, Δt = extension time between d_min and d_max.

Each series of images was measured using a 3D image analysis system (SYNAPSE VINCENT; Fuji Film Medical, Tokyo, Japan). An oblique plane through the long-axis dimension of the gastric antrum was obtained to measure gastric motility. Gastric motility was quantified based on the amplitude and velocity of the peristaltic wave with the smallest antrum diameter. Amplitude was calculated as the difference between the maximum and minimum antrum diameters at the point of the smallest diameter in the wave. Velocity was calculated as the extension or contraction velocity of the antrum at the same point. The amplitude and velocity were defined as follows (Fig. 1):

$$\begin{array}{c} \mathrm{Amplitude}\left(\mathrm{mm}\right)\;=\mathrm{Maximum diameter}\left({d\_}_{\max}\right)–\\ \mathrm{Minimum diameter}\left({d\_}_{\min}\right) \end{array}$$

$$\begin{array}{c} \mathrm{Extension or contraction velocity}\left(\mathrm{mm}/s\right)\;=\;\left({d\_}_{\max}{–\;d\_}_{\min}\right)/\Delta t \end{array}$$

$$\begin{array}{c} \Delta t\left(\sec\right)={\mathrm{extension or contraction time between d}\_}_{\min}{\mathrm{and d}\_}_{\max} \end{array}$$

A sagittal plane through the long-axis dimension of the duodenal second portion was obtained to measure duodenal motility. Duodenal motility was evaluated at the point with the smallest diameter and was quantified by measuring the difference between maximum and minimum diameters during the scanning time.

**Fig. 2.**
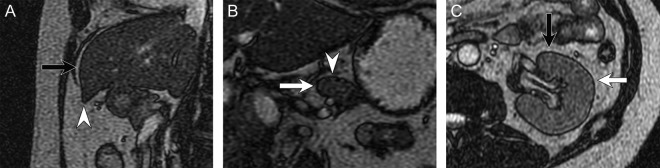
Examples of measurement points for abdominal organ motion. (**A)** Left lobe of the liver in the sagittal image. (**B**) Pancreatic body and tail in the coronal image. (**C)** Left kidney in the axial image. Arrows indicate the measurement points of organ edges. White arrow, left–right direction; black arrow, antero–posterior direction; white arrowhead. caudal–cranial direction.

**Fig. 3.**
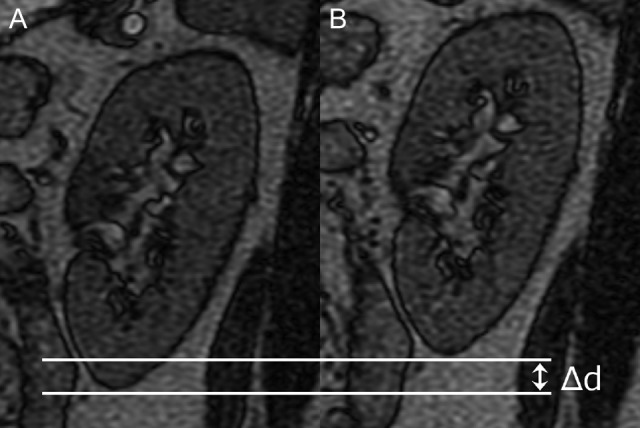
An example of the measurement for maximum displacement (Δd) of the caudal–cranial direction of the left kidney in the sagittal image. (**A)** The most caudal position of the left kidney. (**B)** The most cranial position of the left kidney.

Abdominal organ motions were measured at the left and right lobes of the liver; pancreatic head, body and tail; and both kidneys. Three orthogonal planes were selected for the measurement of the respective organ regions: left lobe of the liver—the proximal bifurcation of the lateral branch of the left portal vein, the right lobe of the liver—the first main bifurcation of the right portal vein; the pancreatic head, body and tail—the center of each structure; both kidneys—the center of each structure. Abdominal organ motions were quantified by calculating the maximal displacement of the organ edges in the left–right (LR), antero–posterior (AP), and caudal–cranial (CC) directions on all three planes (Figs 2 and 3). Clearly identifiable positions of abdominal organs were selected for the measurements, and we measured the same points of each organ in the fasting and postprandial states. All measurements were performed with the consensus by two radiologists.

### Statistical analysis

The Wilcoxon signed-rank test was used for statistical analysis. A test value of *P* < 0.05 was considered statistically significant. All statistical analyses were performed using JMP version 13.0.0 (SAS Institute Inc., Cary, NC, USA).

## RESULTS

**Fig. 4.**
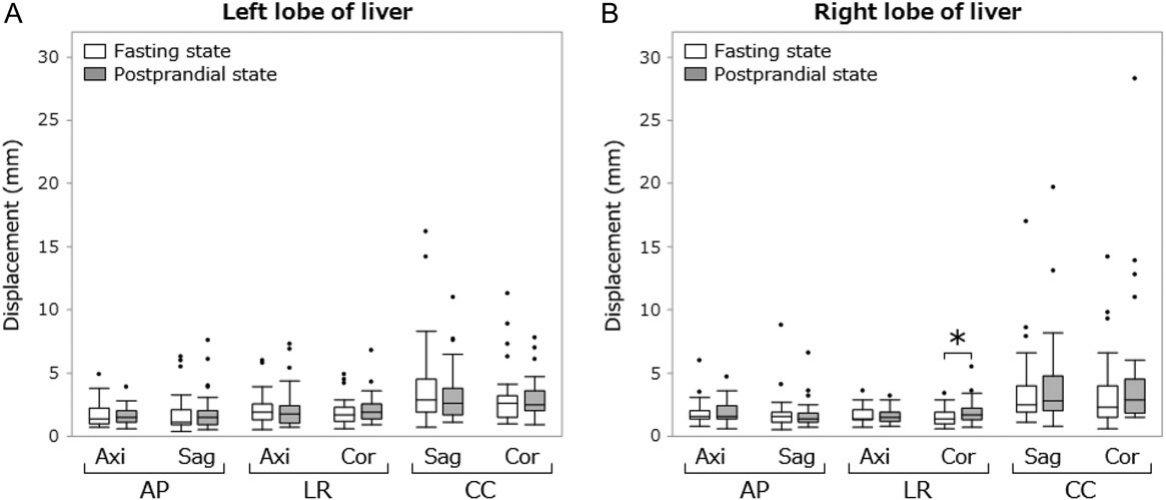
Box plot of displacements of the liver. The bottom and top of each box indicate the first and third quartiles; the bar inside the box indicates the median. Upper and lower whiskers show the maximum and minimum values within 1.5 interquartile measurements from the box, respectively. The black points indicate outliers. The asterisk indicates *P* < 0.05. AP = antero-posterior, LR = left-right, CC = caudal-cranial, Axi = axial plane, Sag = sagittal plane, Cor = coronal plane.

**Fig. 5.**
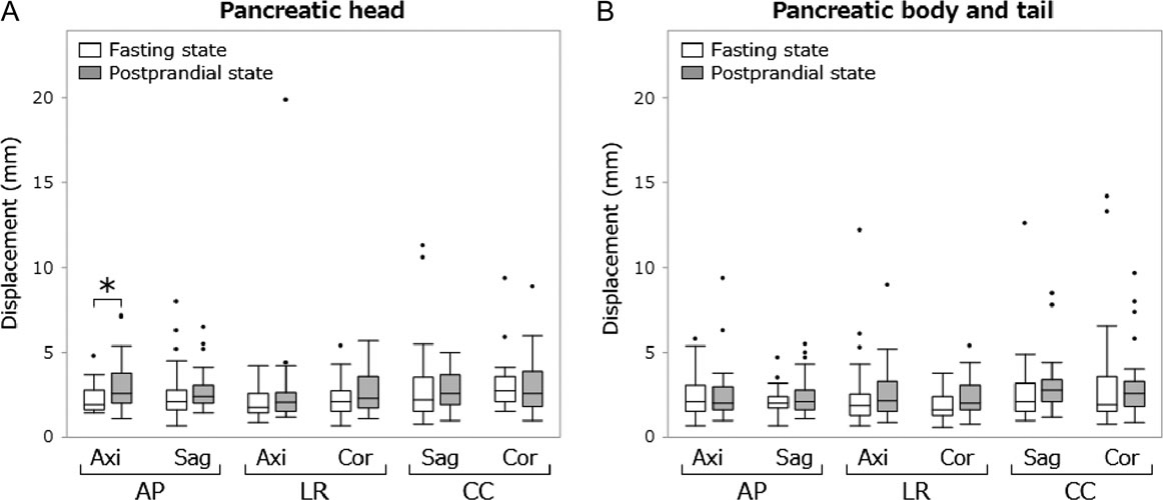
Box plot of displacements of the pancreas. The bottom and top of each box indicate the first and third quartiles; the bar inside the box indicates the median. Upper and lower whiskers show the maximum and minimum values within 1.5 interquartile measurements from the box, respectively. The black points indicate outliers. The asterisk indicates *P* < 0.05. AP = antero-posterior, LR = left-right, CC = caudal-cranial, Axi = axial plane, Sag = sagittal plane, Cor = coronal plane.

**Fig. 6.**
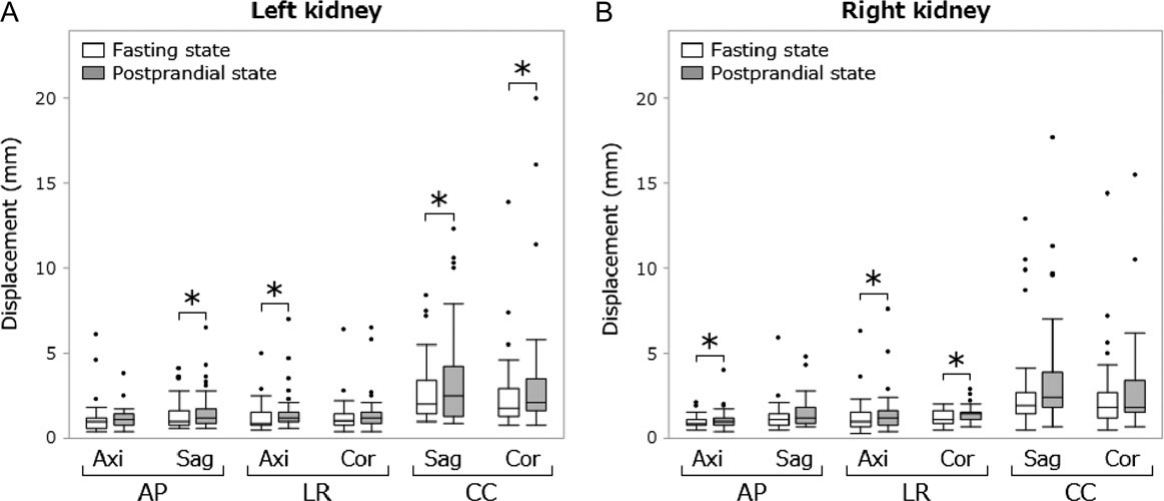
Box plot of displacements of the kidneys. The bottom and top of each box indicate the first and third quartiles; the bar inside the box indicates the median. Upper and lower whiskers show the maximum and minimum values within 1.5 interquartile measurements from the box, respectively. The black points indicate outliers. The asterisks indicate *P* < 0.05. AP = antero-posterior, LR = left-right, CC = caudal-cranial, Axi = axial plane, Sag = sagittal plane, Cor = coronal plane.

The median amplitudes of the antral peristaltic wave in the fasting and postprandial states were 7.7 mm (range, 1.5–27.7) and 15.1 mm (range, 2.9–35.1), respectively (*P* < 0.01). The median velocities of the wave in the fasting and postprandial states were 1.3 mm/s (range, 0.2–3.5) and 2.5 mm/s (range, 0.7–4.2), respectively (*P* < 0.01). The median differences between the maximal and minimal duodenum diameters were 7.5 mm (range, 2.6–12.9) for the fasting state and 9.4 mm (range, 2.9–20.5) for the postprandial state (*P* = 0.07). Median abdominal organ displacements in the two states are shown in Table 1. Box plots of abdominal organ displacements are shown in Figs 4–6. The median displacements of all organs in three directions in the fasting and postprandial states ranged from 0.9 to 2.9 mm (median, 1.8), and from 1.0 to 2.9 mm (median, 2.0), respectively. Significant increases in organ motion in the postprandial state were observed within the following organs. Right lobe of the liver—LR direction on the coronal plane: 1.4–1.7 mm; pancreatic head—AP on axial: 1.9–2.6 mm; left kidney—AP on sagittal: 1.0–1.2 mm, LR on axial: 0.9–1.2 mm, CC on sagittal: 2.0–2.5 mm, CC on coronal: 1.8–2.1 mm; right kidney—AP on axial: 0.9–1.0 mm, LR on axial: 1.0–1.2 mm, LR on coronal: 1.1–1.4 mm.

**Table 1.** Median displacements of abdominal organs

Organs	Median displacements, mm (range)					
	Anterior–posterior		Left–right		Caudal–cranial	
	Axial	Sagittal	Axial	Coronal	Sagittal	Coronal
Left lobe of the liver						
Fasting	1.4 (0.7–4.9)	1.1 (0.4–6.3)	1.9 (0.5–6.0)	1.7 (0.6–4.9)	2.9 (0.7–16.2)	2.6 (1.0–11.3)
Postprandial	1.5 (0.6–3.9)	1.5 (0.5–7.6)	1.8 (0.7–7.3)	1.9 (0.9–6.8)	2.6 (1.1–11.0)	2.5 (0.9–7.8)
*P*-value	0.57	0.52	0.47	0.22	0.59	0.69
Right lobe of the liver						
Fasting	1.6 (0.8–6.0)	1.6 (0.5–8.8)	1.4 (0.7–3.6)	1.4 (0.6–3.4)	2.5 (1.1–17.0)	2.3 (0.6–14.2)
Postprandial	1.6 (0.6–4.7)	1.4 (0.7–6.6)	1.5 (0.8–3.2)	1.7 (0.7–5.5)	2.8 (0.8–19.7)	2.9 (1.5–28.3)
*P*-value	0.38	0.69	0.81	0.04	0.55	0.39
Pancreatic head						
Fasting	1.9 (1.4–4.8)	2.1 (0.7–8.0)	1.8 (0.9–4.2)	2.1 (0.7–5.4)	2.2 (0.8–11.3)	2.8 (1.5–9.4)
Postprandial	2.6 (1.1–7.2)	2.4 (1.4–6.5)	2.1 (1.2–19.9)	2.3 (1.1–5.7)	2.6 (1.0–5.0)	2.6 (1.0–8.9)
*P*-value	<0.01	0.09	0.23	0.11	0.12	0.71
Pancreatic body and tail						
Fasting	2.1 (0.7–5.8)	2.0 (0.7–4.7)	1.9 (0.7–12.2)	1.6 (0.6–3.8)	2.1 (1.0–12.6)	1.9 (0.8–14.2)
Postprandial	2.0 (1.0–9.4)	2.1 (1.1–5.5)	2.2 (0.9–9.0)	2.0 (0.8–5.4)	2.8 (1.2–8.5)	2.6 (0.9–9.7)
*P*-value	0.82	0.32	0.5	0.05	0.15	0.32
Left kidney						
Fasting	1.0 (0.4–6.1)	1.0 (0.6–4.1)	0.9 (0.5–5.0)	1.1 (0.4–6.4)	2.0 (1.0–8.4)	1.8 (0.8–13.9)
Postprandial	1.1 (0.4–3.8)	1.2 (0.6–6.5)	1.2 (0.6–7.0)	1.2 (0.4–6.5)	2.5 (0.9–12.3)	2.1 (0.8–20.0)
*P*-value	0.17	0.01	<0.01	0.11	0.02	0.03
Right kidney						
Fasting	0.9 (0.5–2.1)	1.1 (0.5–5.9)	1.0 (0.3–6.3)	1.1 (0.5–2.0)	1.9 (0.5–12.9)	1.8 (0.5–14.4)
Postprandial	1.0 (0.4–4.0)	1.2 (0.7–4.8)	1.2 (0.4–7.6)	1.4 (0.7–2.9)	2.4 (0.7–17.7)	1.8 (0.7–15.5)
*P*-value	0.04	0.12	0.04	0.03	0.14	0.33

## DISCUSSION

During and after food intake, the proximal stomach relaxes and serves initially as a reservoir for a large amount of food, and then pushes the food distally through tonic contractions. Simultaneously, powerful and regular peristaltic contractions in the distal stomach mix and grind the food, while it moves to the duodenum [14].

In the present study, the amplitude and velocity of the antrum peristaltic wave in the postprandial state increased significantly compared with those in the fasting state; moreover, increased gastric motility was confirmed at the gastric antrum in the postprandial state. Teramoto *et al.* [10] studied duodenal motility after the ingestion of a liquid meal in healthy volunteers using cine-MRI; they found that the shift in the center of gravity of the duodenum and the velocity of duodenal wall motion were both smallest immediately after ingestion, and then increased significantly in the subsequent 30 and 60 min, respectively. However, significant differences in duodenal motility were not observed between fasting and postprandial states in our study. We analyzed duodenal motility as only the difference between the maximum and minimum duodenal diameters. The difference in the evaluation methods for duodenal motility between the two studies might be the cause of the conflicting results.

Significant increases in abdominal organ displacements in the postprandial state were observed in the following organs: the right lobe of the liver in the LR direction, the pancreas head in the AP direction, the left kidney in the three directions, and the right kidney in the AP and LR directions. The displacements of the left kidney (in the CC direction) and the right kidney (in the LR direction) exhibited significant increases on two planes; other displacements exhibited increases in motion on only one plane. Due to the irregular organ shape and trajectory, one plane alone could not completely detect abdominal organ motion, which might have led to the discrepancy between two planes. Although significant increases were observed in several abdominal organ displacements, the differences in median abdominal displacements between the fasting and postprandial states were <1 mm.

Wysocka *et al.* [15] analyzed gastric motion in the fasting and postprandial state with 10 healthy volunteers, using cine-MRI with a 30-second breathing hold. The median of mean gastric displacements from the baseline position was small, rarely exceeding 1.1 mm, and the median standard deviation of the displacements was also small (range, 2.1–3.6 mm) in the fasting and postprandial states. Both the displacements and the standard deviations did not differ significantly between the two states. The authors concluded that non-respiratory intra-fractional gastric motion was small, and that gastric position was stable after small and standard meals. Although we measured gastric motility at the antrum using cine-MRI, similarly to previous studies [6–8], Wysocka *et al.* [15] measured it with the gastric outline on the axial, coronal, and oblique planes. Taking their findings into account, it seems reasonable that in our study, abdominal organ displacements increased by a small margin in the postprandial state; this may be attributed to the possibility of a minimal difference in abdominal organ motion induced by gastric motion between the fasting and postprandial states.

Kirilova *et al.* [12] used cine-MRI to measure the motion of liver tumors during free breathing, and found that the average CC tumor motion was 15.5 mm, AP motion was 10.1 mm and LR motion was 7.5 mm. Moerland *et al.* [16] studied respiration-induced motion of the kidneys using MRI. Displacements in a tilted coronal plane through the longitudinal axis of the kidneys under normal respiration conditions were 2–24 mm in the left kidney and 4–35 mm in the right kidney; furthermore, displacements under forced respiration conditions were much larger in the left and right kidneys at 10–66 mm and 10–86 mm, respectively. Heerkens *et al.* [13] used cine-MRI to evaluate the pancreatic tumor motion during breathing; the average CC tumor motion was 15 mm, AP motion was 5 mm and LR motion was 3 mm. Compared with these organ or tumor motions that were mainly induced by respiration, the differences in abdominal organ displacement between the fasting and postprandial states in our study were quite small.

Consequently, it can be seen that the abdominal organ displacements under different conditions of fasting and postprandial states are quite small. Although we should add a margin for the intra-fractional abdominal organ motion itself, it is not necessary to take changes in the intra-fractional abdominal organ motions in the two different states into consideration in most treatment planning or irradiation for these organs. That is, strict management such as fasting before irradiation or large expansion of the internal margin in planning, does not appear to be necessary for the management of the intra-fractional abdominal organ motion changes under different gastric motilities in the two states.

Our study had some limitations. First, cine-MRI was performed with breath holding to eliminate abdominal organ motion induced by respiration; however, respiratory motion of these organs was not completely eliminated. We found that 35 of all measured abdominal organ displacements exceeded 10 mm, of which 33 were in the CC direction. Notably, we observed that every organ with displacements >10 mm in the CC direction moved synchronously with the diaphragm. Although several organ displacements were quite large, most of them were in the CC direction, which was likely respiratory motion caused by the failure of subjects to hold their breath. However, abdominal organ motion in our study with breath holding was substantially smaller than respiratory motion of abdominal organs or tumors in previous studies [12, 13, 16]. Therefore, while we could not eliminate respiratory motions of these organs completely, these motions were minimized compared with previous breathing studies. Second, other internal organ motions (i.e., cardiac beats and small and large intestine activities) were also not eliminated in our study. However, it is difficult to evaluate the abdominal organ motion induced by gastroduodenal peristaltic motion separately from the organ motion induced by other internal activities.

Third, we did not observe a significant increase in duodenal motility. We had planned to quantify duodenal motility based on both the velocity and amplitude of the duodenal peristaltic wave, but we could not obtain images clear enough to smoothly visualize peristaltic motion as a series of waves. Therefore, we only quantified the difference between the maximum and minimum diameter of the duodenum. Although our measurement method was not enough to assess the change in duodenal motility, taking Teramoto’s reports [10] and the *P* value of our analysis in duodenal motility into consideration, it is probable that duodenum motility actually increased in the 35 healthy volunteers after injection.

Fourth, further analyses with real tumor displacement may be required to reveal the uncertainty in intra-fractional abdominal tumor motion that is induced by gastrointestinal motility.

## CONCLUSIONS

Although the motion of several abdominal organs increased in the postprandial state, the differences in abdominal organ displacement between the fasting and postprandial states were minimal. Therefore, our study suggests that it is not necessary to strictly manage gastric conditions, or to add excessive margins in most treatment planning and irradiation for the intra-fractional abdominal organ motion changes under different gastric motilities in the fasting and postprandial states.

## ACKNOWLEDGEMENTS

We thank Dr. Jin Yamamoto for his support. We presented this study with a slightly different analysis in a smaller number of volunteers at the 59th Annual Meeting of American Society for Radiation Oncology in 2017.

## CONFLICT OF INTEREST

The authors state that there are no conflicts of interest.
